# The Degree of Influence of Daily Physical Activity on Quality of Life in Type 2 Diabetics

**DOI:** 10.3389/fpsyg.2020.01292

**Published:** 2020-06-30

**Authors:** Feng Zhang, Li Huang, Li Peng

**Affiliations:** ^1^College of Physical Education, Southwest University, Chongqing, China; ^2^Key Lab of Physical Fitness Evaluation and Motor Function Monitoring, Southwest University, Chongqing, China

**Keywords:** physical activity, quality of life, influence factor, T2DM, dimension

## Abstract

**Objectives:** This study mainly explored the degree of influence of daily physical activity (PA) on the quality of life (QoL) and then provided a basis for improving PA and QoL in patients with Type 2 diabetes mellitus (T2DM).

**Methods:** Long International PA Questionnaire (IPAQ-L) and Diabetic Mellitus QoL Scale (DMQLS) were used to investigate the daily PA and QoL, respectively, of patients with T2DM. The obtained data were analyzed by SPSS19.0 data analysis software. The correlation between PA and QoL was analyzed by Pearson correlation analysis and the degree of influence of PA on the QoL of patients with T2DM was analyzed by optimal scale regression analysis.

**Results:** The total daily PA of the participants was 1172.79 ± 1266.23 MET-min/week and the overall score of QoL was 149.17 ± 17.16 points. Positive correlation has been found between PA and QoL and all its dimensions (all *P* < 0.05). It was found in the optimal scale regression that among many influencing factors, PA was significant and ranks first in importance among factors of the patient’s total QoL (importance = 0.432, *P* < 0.05) and its disease, physiological, psychological, and social dimension. The degrees of influence of PA on sub-dimensions of QoL were in the following order: physiology (0.476), society (0.441), disease (0.388), psychology (0.377), and satisfaction (0.089).

**Conclusion:** The level of daily PA of patients with T2DM in southwestern China was moderate. Among the influencing factors such as gender, age, course of disease, education background, monthly income, BMI, work status, and complications, daily PA is the most important factor for QoL and its physiological, social, psychological, and disease dimension. The degrees of influence of daily PA on sub-dimensions of QoL were (in descending order) as follows: physiology, social, disease, psychology, and satisfaction. Therefore, in view of the importance of daily PA for QoL, more attention should be paid to the daily PA of T2DM patients in the future, including the study on more targeted testing methods, promotion strategies of daily PA, and its more extensive positive effects.

## Introduction

As early as 2009, the famous American sports epidemiologist Blair (2009) pointed out that insufficient physical activity (PA) would be the biggest public health problem in the 21st century. In 2010, the World Health Organization (WHO) ranked insufficient PA as the fourth most significant risk factor for human death (World Health Organization [WHO], 2010). A large number of epidemiological studies of PA and the “Guidelines for PA Promotion” in various countries also prove that exercise is an important way to prevent and treat diseases (Chinese Medical Association Diabetes Branch, 2017). Diabetes is a group of metabolic diseases characterized by chronically elevated blood glucose levels and has become the third largest disease after cancer and cerebrovascular disease with the highest morbidity and mortality in the world. The prevalence of Type 2 diabetes mellitus (T2DM) among the world’s adults is expected to increase to 4.4% by 2030, and the number of T2DM worldwide is expected to increase from 200 million to about 400 million, reaching 592 million by 2035 (Shi and Hu, 2014). As of 2018, the prevalence of diabetes in China has reached 11.6%, ranking the first in the world. According to WHO, the number of T2DM patients in China will rise to 380 million by 2025.

The pathogenesis and etiology are not clear, and there is no cure for T2DM. However, some studies have shown that the PA of diabetic patients is significantly lower than that of normal people (Kumar et al., 2016), and low PA or lack of exercise has been proven to be closely related to the occurrence and development of chronic diseases such as type II diabetes. China’s “Guidelines for the Prevention and Treatment of T2DM” stated that insufficient PA is one of the important factors leading to the rapid increase in the number of T2DM in China (Chinese Medical Association Diabetes Branch, 2017). The incidence of mortality and cardiovascular events of T2DM were lower in the high PA group (Hidekatsu et al., 2018). High-intensity training ameliorates metabolic parameters in individuals at risk of or with type 2 diabetes (Jelleyman et al., 2015) and that low PA doubles an increased risk of incident type 2 diabetes as compared with high PA (Cloostermans et al., 2015). “Health China 2030 program outline” proposed to further strengthen the integration of sports and medical, non-medical health intervention to control the health threat of unhealthy lifestyle diseases policy. At the same time, it mentioned the creation of a new model of national fitness, which combines physical medicine with national fitness, adhering to the basic standards of national fitness performance based on entertainment, extensiveness, competition, and sociality; the public policy of national fitness should be carried out on the basis of improving and optimizing the physical quality of all grassroots people, controlling the incidence of chronic diseases, and improving the survival rate of chronic diseases.

World Health Organization lists health-related quality of life (QoL) as a new generation of health assessment indicators, defined as how individuals in different cultures and value systems feel about their place in life, and experiences related to the state of life including the life goals, expectations, standards, and concerns, being divided into four dimensions, namely, the physical, psychological, social functions, and physical states (WHOQOL Group, 1993). It is worth noting that most of the studies on patients with diabetes used the SF-36 scale (36 concise health status questionnaire), which is widely used in the study of chronic diseases, but not specific enough for patients with diabetes. In China, the QoL scale was developed for diabetic patients in 1997, which measured the four dimensions of life, heart, society, and treatment; in 2005, a special tool was used for evaluating the QoL of diabetic patients, which is developed from the dimensions of disease, physiology, psychology, society, and satisfaction in 87 items; Fu et al. (2010) simplified the QoL scale of 2005, from 87 items to 39 items according to Chinese national conditions, and made it more effective and kept its dimension unchanged. A large number of studies have shown that the QoL of patients with T2DM is significantly lower than that of healthy people (Schulze and Hu, 2005; Alshayban and Royes, 2020). PA has closed relationship with QoL (Nana et al., 2012). A study was conducted in adults with T2DM in Canada, showing a significant association between PA and health-related QoL (Thiel et al., 2017). An observational study in Sweden demonstrated that PA and QoL in people who received PA as a prescription improved during a follow-up period of 2 years (Rödjer et al., 2016). At the same time, some authors believed that PA level should be used as one of the important indicators to measure the QoL of the elderly (Xue, 2010).

In summary, since it seems impossible to find a cure in a short period of time, it is imperative to reduce the pain and improve the QoL of such a huge number of Chinese diabetics. However, the degree of influence of PA on QoL and its different dimensions of patients withT2DM are not clear. Therefore, the purpose of this study was to investigate the PA and QoL of patients with T2DM, and we tried to analyze the degree of influence of PA on QoL and its sub-dimensions.

## Subjects and Methods

### Subjects

Patients are 1-week outpatient cases from hospitals in three provincial capitals in Southwest China. Inclusion criteria are as follows: (1) conforming to the 1999 diagnostic criteria for diabetes, non-insulin-dependent; (2) no disorders in motor system or other serious diseases that affect PA; and (3) consciously aware and no difficulty in communicating with investigators. Exclusion criteria are as follows:(1) other types of diabetics; (2) pre-diabetes; (3) less than 30 days of diagnosis; (4) combined with progressive or systemic disease or mental illness or during pregnancy or lactation; (5) unable to express their emotions; (6) impaired PA; and (7) age under 18 years old.

### Methods

#### Questionnaire

The questionnaire consists of three components: (1) Patient Basic Information Collection, (2) Diabetic Mellitus QoL Scale (DMQLS), and (3) The Long International PA Questionnaire (IPAQ-L). The patient received the questionnaires and filled them in on-site, and a trained research assistant was present to assist participants with the questionnaire. A total of 270 questionnaires were distributed and 255 valid questionnaires were finally obtained, with an efficiency of 94%.

#### Patient Basic Information Questionnaire

Patients were surveyed on gender, age, course of disease, education, monthly income, height, weight, and complications, etc. (Table 1).

**TABLE 1:** Basic information of the samples (*N* = 255).

Variable		*n*	Percentage (%)
Gender	Male	126	49.4
	Female	129	50.6
Age	<44	17	6.7
	45–59	83	32.5
	>60	155	60.8
Educational background	Primary school	42	16.5
	Junior high school	81	31.8
	High school	72	28.2
	College	60	23.5
Work	Have	67	26.3
	Haven’t	188	73.7
Monthly income	<2000 Yuan	56	22.0
	2000–5000 Yuan	174	68.2
	>5000 Yuan	25	9.8
Complications	Have	186	72.9
	Haven’t	69	27.1
Course of disease	<1 year	12	4.7
	1–5 years	102	40.0
	>5 years	141	55.3

#### Diabetic Mellitus QoL Scale (DMQLS)

The revised DMQLS was used to measure the QoL of patients with T2DM. The questionnaire involved 39 items including five dimensions: disease dimension (13 items), physiological dimension (7 items), psychological dimension (6 items), social dimension (6 items), and satisfaction dimension (7 items; Fu et al., 2010). The reliability of this questionnaire is 0.924. The coefficient values of each dimension were 0.767, 0.689, 0.920, 0.908, and 0.854, respectively, all greater than 0.6, indicating that the questionnaire was credible. This questionnaire is scored on a five-point scale. The higher the score is, the better the QoL will be.

#### Long International PA Questionnaire (IPAQ-L)

The IPAQ questionnaire is an international PA questionnaire developed by the International Consensus Group in 1997. Qu and Keji (2004) translated the IPAQ-L into a Chinese version and measured all the coefficient values greater than 0.7, indicating that the questionnaire has good reliability and validity. It is confirmed that the IPAQ-L is effective in testing Chinese PA (Duncan et al., 2011). The questionnaire consisted of five parts: job-related PA, housework-related PA, transportation PA, sports and leisure-time PA, and sitting time. Each aspect of PA consists of three levels of activity with a total of 13 items. Each item needs to fill in the activity time (not less than 10 min each time) and the frequency of PA in a week. According to the energy consumption valuation of each PA obtained by the survey, the total energy consumption of the PA of the subject for seven consecutive days is calculated, that is, the Metabolic equivalent (MET) of different types of PA × activity time (min) × frequency per week = total energy expenditure (MET-min) of patients for 7 consecutive days. The PA level of the respondents was divided into three groups: PA deficiency group, moderate PA group, and high PA group (Mengyu et al., 2014).

### Statistical Analysis

Data obtained in this study were analyzed using SPSS 19.0. Descriptive analysis of basic information, QoL, and PA of patients with T2DM and expressed as means ± standard deviation or percentage. The correlation between PA and QoL was analyzed by Pearson correlation analysis and the degree of influence of PA on the QoL of patients with T2DM was analyzed by Optimal Scale Regression analysis. The significance level of all variables was set as α = 0.05.

## Results

### PA and QoL in Patients With T2DM

According to the results from IPAQ-L, 255 patients were sorted into three groups: a maximum of 177 patients in the moderate PA group, accounting for 69.41%; 42 patients in the high PA group, accounting for 16.47%; and only 36 patients with insufficient PA, accounting for 14.11%. The total volume of PA was 1712.79 ± 1266.23 MET-min, generally at the moderate level. As for the contribution to overall PA: leisure-time PA ranked the first with 692.17 ± 686.58 MET-min, housework PA ranked the second with 404.60 ± 562.97 MET-min, job-related PA ranked the third with 312.82 ± 644.21 MET-min, and the fourth one was transportation PA with 310.01 ± 365.48 MET-min (Table 2). The total average score of QoL was 149.17 ± 17.16 points. The score in sub-dimension was as follows: disease dimension 50.6 ± 5.52 points, physiological dimension 26.46 ± 3.73 points, social dimension 24.00 ± 5.06 points, psychological dimension 24.94 ± 4.24 points, and satisfaction dimension 23.16 ± 4.06 points (Table 3).

**TABLE 2:** Daily PA in patients with T2DM.

Group dimension	Overall	High	Moderate	Deficient
N (%)	255(100%)	42(16.47%)	177(69.41%)	36(14.11%)
Job	312.82 ± 644.21	945.02 ± 1127.89	212.85 ± 421.00	56.88 ± 129.45
Transportation	310.01 ± 365.48	414.35 ± 488.16	337.72 ± 344.41	52.08 ± 91.41
housework	404.60 ± 562.97	1100.95 ± 819.95	304.02 ± 388.57	86.67 ± 127.26
Leisure time	692.17 ± 686.58	1599.98 ± 950.02	563.73 ± 459.65	264.57 ± 201.98
Total	1712.79 ± 1266.23	4007.92 ± 1299.15	1423.02 ± 504.74	459.84 ± 129.90
Sitting time	1315.64 ± 758.37	1018.03 ± 600.65	1354.74 ± 742.67	1470.62 ± 915.87

PA: MET*min/week; sitting time: min/week.

**TABLE 3:** QoL in patients with T2DM.

	Disease dimension	Physiological dimension	Psychological dimension	Social dimension	Satisfaction dimension	Total
Score	50.6 ± 5.52	26.46 ± 3.73	24.94 ± 4.24	24.00 ± 5.06	23.16 ± 4.06	149.17 ± 17.16

### Correlation Between PA and QoL of Patients With T2DM

Positive correlation (Table 4) has been found between PA and QoL and all its sub-dimensions (all *P* < 0.05). Psychological and satisfaction dimensions of QoL were only lowly correlated with PA (*r*_*psychological dimension*_ = 0.146, *r*_*satisfaction dimension*_ = 0.290, and both *r* < 0.3).

**TABLE 4:** Pearson correlation between PA and QoL.

		PA	QoL	Disease dimension	Physiological dimension	Psychological dimension	Social dimension	Satisfaction dimension
PA	*r*	1						
QoL	*r*	0.394**	1					
Disease dimension	*r*	0.335**	0.859**	1				
Physiological dimension	*r*	0.311**	0.728**	0.445**	1			
Psychological dimension	*r*	0.146*	0.730**	0.537**	0.417**	1		
Social dimension	*r*	0.383**	0.784**	0.575**	0.684**	0.414**	1	
Satisfaction dimension	*r*	0.290**	0.648**	0.580**	0.264**	0.409**	0.222**	1

*P < 0.05, **P < 0.01.

The total score of QoL and the scores of sub-dimensions in patients with T2DM were used as dependent variables; sex, age, education, monthly income, years of illness, complications, job status, body mass index, and PA level were used as independent variables for optimal scale regression analysis. When performed for the overall score of QoL of patients with T2DM, the model has statistical significance (*F* = 10.27, *P* < 0.05, and adjusted *R*^2^ = 0.354). Therefore, it can be determined that the relationship between the independent variables (gender, age, education, monthly income, work status, body mass index, and PA level), which entered the equation and the overall QoL score, was statistically significant (*P* < 0.05). Examination of the correlation and tolerance of the individual variables with the total QoL scores revealed that the tolerances after conversion of each variable were above 0.6, indicating that there is no strong collinear relationship between the independent variables of the equation. Based on their importance, the ranking of these independent variables are as follows: PA level (0.432), education (0.152), work status (0.102), body mass index (0.087), complications (0.085), monthly income (0.072), course of illness (0.046), age (0.020), and gender (0.005; Table 5).

**TABLE 5:** Optimal regression analysis of PA on total QoL score.

Independent variable	Assignment	*B*	*F*	*P*	Importance
Gender	1 = male 2 = female	0.137	5.413	0.021	0.005
Age	1 = Under 44 2 = 45–59 3 = Above 60	0.075	3.065	0.048	0.020
Education	1 = Primary school 2 = Junior high school 3 = High school 4 = College	0.188	9.489	0.000	0.152
Monthly income	1 = Under 2000 Yuan 2 = 2000–5000 Yuan 3 = Above 5000 Yuan	0.183	11.637	0.000	0.072
Disease course	1 = Under 1 year 2 = 1–5 year 3 = Above 5 year	0.091	4.06	0.018	0.046
Complications	1 = Have 2 = Haven’t	0.105	4.229	0.041	0.085
Work	1 = Have 2 = Haven’t	0.141	6.085	0.014	0.102
BMI	1 = underweight 2 = normal 3 = overweight	0.142	7.276	0.001	0.087
PA level	1 = Insufficient 2 = Sufficient	0.368	29.711	0.000	0.432

**FIGURE 1:**
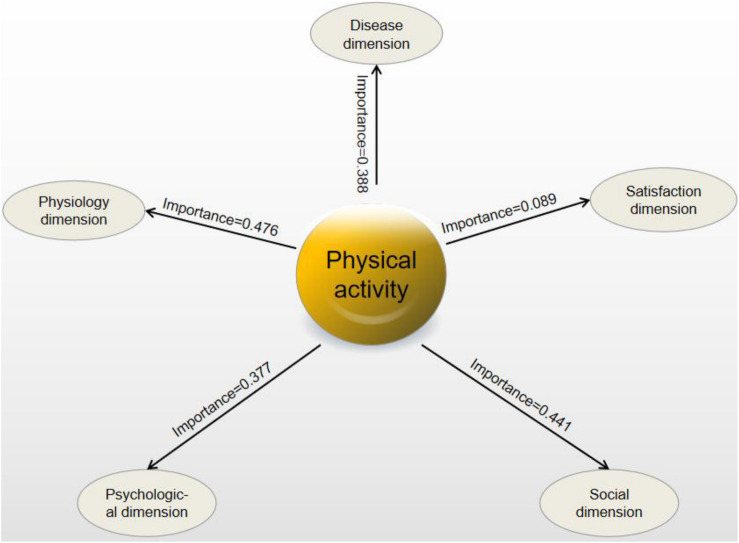
Influence degree of PA on sub-dimensions of QoL.

Similarly, when performing the optimal regression analysis for disease (*F* = 7.914, *P* < 0.05, and adjusted *R*^2^ = 0.290), physiological (*F* = 5.514, *P* < 0.05, and adjusted *R*^2^ = 0.210), psychological (*F* = 3.532, *P* < 0.05, and adjusted *R*^2^ = 0.130), social (*F* = 9.607, *P* < 0.05, and adjusted *R*^2^ = 0.337), and satisfaction (*F* = 4.672, *P* < 0.05, and adjusted *R*^2^ = 0.178) dimension scores of patients, all models appeared statistically significant. Except for the satisfaction dimension, PA ranked as the most important factor for QoL and its physiological, social, psychological, and disease dimension among gender, age, course of disease, education background, monthly income, BMI, work status, and complications. The degrees of influence of PA on sub-dimensions of QoL were in the following order: physiology (0.476), society (0.441), disease (0.388), psychology (0.377), and satisfaction (0.089; Figure 1).

## Discussion

### QoL and PA in Patients With T2DM

Under the modern integrative medicine model, promotion of QoL has become the highest goal of all health interventions in all fields of medicine. Studies have pointed out that quantitative measurement of QoL in patients with diabetes not only can know the size of the disease impact on patients but also can find other factors, so that effective intervention measures can finally be applied to patients. The WHO has listed the QoL as a new generation of health assessment indicators, and it was widely used in chronic diseases including evaluation of the physical, psychological, and social functional status of individuals/groups (Zhang and Cai, 2012). QoL has also been used as one of the important indexes to evaluate diabetes, but there is no uniform evaluation tool in the world. There are many researches on the QoL of patients with T2DM, most of which found that the QoL of patients with Type II diabetes was lower than that of normal people. It is worth noting that most of the studies on QoL of diabetes uses the SF-36 scale (36 concise health status questionnaire), which is widely used in the study of chronic diseases, but not specific enough for patients with diabetes. This study used a QoL scale, which is not only specific for diabetic patients, which better reflects the characteristics of some diseases of diabetic patients (Li, 2005), but also specific for Chinese. Fu et al. (2010), using the same scale, found that the QoL score (151.12 ± 18.61 points) of T2DM patients was significantly lower than that of non-patients (186.37 ± 16.53 points). The average score of QoL in middle-aged and elderly community diabetes patients was 119.27 ± 20.442 points, which is in very poor condition (Liu and Sun, 2018). The total scores of QoL of the T2DM patients in this study (149.17 ± 17.16 points) were lower than those in the above study of Fu Chen Chao, but higher than that of Liu.

Insufficient PA has become one of the most important public health problems in the 21st century. More and more researchers have paid attention to PA. This study used the IPAQ-L to investigate the daily PA of patients with T2DM. IPAQ is used as a comparable and standardized self-report measure of habitual PA of populations from different countries and socio-cultural contexts (Craig et al., 2003). Shanghai Ruijin Hospital verified the adaptability of the IPAQ-L in patients with T2DM in China (Chen et al., 2006). The results of this study showed that the total amount of daily PA in patients with T2DM was 1712.79 ± 1266.23 MET-min, at a moderate level, consistent with other studies (Yi and Haiping, 2015; Peng et al., 2017). The PA of the subjects surveyed was in order of their contribution as leisure-time, housework, job related, and transportation PA. The way for daily PA is no longer based on the consumption of job, transportation and housework-related physical activities, but dominated by leisure-time PA. The PA consumption of the respondents was mainly leisure-time related (Graff-Iversen et al., 2007). The PA level of the United States population has been declining in the past 50 years, and its professional transportation and housework-related physical activities have shown a significant decline, while their leisure-time-related PA has not significantly improved (Brownson et al., 2005).

### The Influence of Daily PA on QoL of Patients With T2DM

The results of Pearson correlation analysis showed that the QoL of patients with diabetes and their various sub-dimensions was significantly positively related to daily PA, which coincided with the results of others. Leng et al. (2012) found that the PA of hemodialysis patients was significantly related to their QoL and mainly reflected in physical health, with weak correlation in spirit, which was consistent with the findings of this study. Higher PA levels were related to a higher QoL, and PA can regulate blood lipids and blood pressure, improve vascular endothelial function, increase insulin sensitivity, and improve physical fitness, which was conducive to the control of diabetes (Gopinath et al., 2012). Therefore, it can be said that there is a significant positive correlation between PA and QoL in diabetic patients. PA alone was beneficial to health regardless of its level, and different types of PA have different effects in promoting health (Powell et al., 2011). Research on diabetic patients found that moderate level of PA can significantly reduce the risk of type II diabetes (Xin et al., 2008). This study used optimal scale regression to explore the extent of PA affects the QoL and its dimensions of diabetic patients. The background idea of optimal scaling regression analysis is to assign quantitative values to the categories of each variable, so that standard programs can be used to solve the quantitative variables (Teng, 2014). It is specially used to solve the problem of how to quantify the classification variables when modeling. The basic idea is based on the model framework of hope fitting, on the premise of ensuring the linear relationship between the variables, through a certain method of repeated iteration, to find the best quantitative score for the original classification variables, and use this score to replace the original variables for subsequent analysis, so that, not only regression analysis, but also any analysis method including all the classification independent variables will be applicable, greatly expanding the scope of application of the analytical method. In this way, standard linear regression method can be used to perform regression analysis on the converted variables, so as to obtain an optimal regression equation (Meulman and Heiser, 2009). It was found that in the optimal scale regression, except for satisfaction dimension, PA was significant and ranked the first importance in influencing factors of the total QoL and its disease, physiological, psychological, and social sub-dimension of the patients with T2DM, which proved again that rational PA can improve the QoL of T2DM, just as other studies did. It has been proven that PA can mainly help patients control weight, control blood sugar levels, and reduce the incidence of other cardiovascular diseases. The energy expenditure of all PA was negatively correlated with serum insulin levels, waist circumference, HDL-C, and blood pressure in patients with type 2 diabetes (Hamasaki et al., 2013). Moderate exercise can increase the body’s immunity and improve insulin resistance, so that blood sugar can be effectively controlled to reduce illness and improve QoL (Yi, 2012). As mentioned in the 2017 edition of the Chinese Guidelines for Diabetes Prevention: “PA plays an important role in the comprehensive management of patients with T2DM.” Regular exercise can increase insulin sensitivity, help control blood sugar, reduce cardiovascular risk factors, and reduce weight. The primary prevention effect is significant for people at high risk of diabetes. Therefore, PA has a significant impact on the QoL of patients with diabetes, especially on the physical and disease dimensions of patients. However, the total score of the psychological dimension and the satisfaction dimension of the patient’s QoL had a very low correlation with the daily PA. Daily PA did not rank the first important factor for the satisfaction dimension of QoL. The satisfaction dimension of QoL in this study refers to the patient’s satisfaction with treatment, medical service, cost, and economics. The medical environment and service/management level have an impact on their treatment satisfaction and medical service satisfaction (Zeng et al., 2016). The treatment and medical service satisfaction were mainly influenced by fasting blood glucose, frequency of hypoglycemia, age, time of insulin use, monthly income, whether living alone, and health education, but not by PA of patients (Cuimei, 2017). It seems that PA cannot affect the treatment and medical service, as well as the cost and economic satisfaction. Then, due to the lack of a radical cure, the long course of the disease, many complications, and high cost of treatment and insufficient medical service of T2DM, the patient’s satisfaction can be affected. This may be the reason why daily PA does not have much impact on its satisfaction of QoL.

Although the IPAQ-L used in this study has been proven to have good reliability and validity, it is, after all, a subjective test method. Many new and objective methods to test PA have emerged now, such as wrist-worn ActiGraph acceleration. Therefore, in future research, these new tools can be used to measure the PA of patients, so as to explore the critical point of the impact of PA on QoL.

## Conclusion

Daily PA of patients with T2DM in southwestern China was generally at a moderate level. Among the influencing factors such as gender, age, course of disease, education background, monthly income, BMI, work status, and complications, daily PA ranked the first important factor for QoL and its physiological, social, psychological, and disease dimension. The degrees of influence of daily PA on sub-dimensions of QoL were (in descending order): physiology, social, disease, psychology, and satisfaction. Therefore, in view of the importance of daily PA for QoL, more attention should be paid to the daily PA of T2DM patients in the future, including the study on more targeted testing methods, promotion strategies of daily PA, and its more extensive positive effects.

## Data Availability Statement

The datasets generated for this study are available on request to the corresponding author.

## Ethics Statement

The studies involving human participants were reviewed and approved by Human Research Ethical Committee of Affiliated Hospital of Southwest University. The patients/participants provided their written informed consent to participate in this study.

## Author Contributions

LP was responsible for the conceptualization and methodology, supervision, and funding acquisition. FZ and LH was responsible for the recruition and screening of subjects, the distribution, and collection of questionnaires. FZ was responsible for data statistical processing and thesis writing. All authors contributed to the article and approved the submitted version.

## Conflict of Interest

The authors declare that the research was conducted in the absence of any commercial or financial relationships that could be construed as a potential conflict of interest.
